# Functional Changes of T-Cell Subsets with Age and CMV Infection

**DOI:** 10.3390/ijms22189973

**Published:** 2021-09-15

**Authors:** Fakhri Hassouneh, David Goldeck, Alejandra Pera, Diana van Heemst, P. Eline Slagboom, Graham Pawelec, Rafael Solana

**Affiliations:** 1 GC01—Immunology and Allergy Group, Maimonides Institute for Biomedical Research of Cordoba (IMIBIC), 14004 Cordoba, Spain; hassounehfakhri@yahoo.com (F.H.); alejandra.pera@imibic.org (A.P.); 2 Department of Internal Medicine II, Centre for Medical Research, University of Tübingen, 72072 Tübingen, Germany; dgoldeck@yahoo.de; 3 Department of Cell Biology, Physiology and Immunology, University of Córdoba, 14004 Cordoba, Spain; 4 Section Gerontology and Geriatrics, Department of Internal Medicine, Leiden University Medical Center, 2333 Leiden, The Netherlands; d.van_heemst@lumc.nl; 5 Molecular Epidemiology, Department of Biomedical Data Sciences, Leiden University Medical Center, 2333 Leiden, The Netherlands; p.slagboom@lumc.nl; 6 Max Planck Institute for Biology of Ageing, 50931 Cologne, Germany; 7 Department of Immunology, University of Tübingen, 72027 Tübingen, Germany; graham.pawelec@uni-tuebingen.de; 8 Health Sciences North Research Institute, Sudbury, ON P3E 2H3, Canada; 9 Immunology and Allergy Service, Reina Sofia University Hospital, 14004 Cordoba, Spain

**Keywords:** aging, cytomegalovirus, CD57, T-cell response

## Abstract

Cytomegalovirus (CMV) latent infection and aging contribute to alterations in the function and phenotype of the T-cell pool. We have demonstrated that CMV-seropositivity is associated with the expansion of polyfunctional CD57+ T-cells in young and middle-aged individuals in response to different stimuli. Here, we expand our results on the effects of age and CMV infection on T-cell functionality in a cohort of healthy middle-aged and older individuals stratified by CMV serostatus. Specifically, we studied the polyfunctional responses (degranulation, IFN-γ and TNF-α production) of CD4+, CD8+, CD8+CD56+ (NKT-like), and CD4−CD8− (DN) T-cells according to CD57 expression in response to Staphylococcal Enterotoxin B (SEB). Our results show that CD57 expression by T-cells is not only a hallmark of CMV infection in young individuals but also at older ages. CD57+ T-cells are more polyfunctional than CD57− T-cells regardless of age. CMV-seronegative individuals have no or a very low percentages of cytotoxic CD4+ T-cells (CD1017a+) and CD4+CD57+ T-cells, supporting the notion that the expansion of these T-cells only occurs in the context of CMV infection. There was a functional shift in T-cells associated with CMV seropositivity, except in the NKT-like subset. Here, we show that the effect of CMV infection and age differ among T-cell subsets and that CMV is the major driving force for the expansion of highly polyfunctional CD57+ T-cells, emphasizing the necessity of considering CMV serology in any study of immunosenescence.

## 1. Introduction

The human immune system evolved to protect and defend the organism against disease and potentially to protect the symbiotic gut microbiota. T-cells are a major component of adaptive immunity, with a high degree of specificity in response to a pathogen challenge, enabling the host to mount a specific immune response and generate immunological memory [1]. Therefore, for effective immune protection against the primary and subsequent challenges, these cells must be maintained in a unimpaired state and appropriately regulated [2]. However, on aging, the immune system undergoes profound changes, loosely termed immunosenescence, that can affect the outcome of the immune response [3]. The impact of these age-related changes on the immune system has been associated clinically with decreased efficacy of vaccines, an increase in the frequency and severity of infectious diseases, and an increased incidence of chronic inflammatory disorders [3,4]. These alterations are associated with phenotypical and functional changes affecting a variety of immune cells, especially T-cells [5,6]. It has been shown that chronic stimulation of the immune system, such as by persistent viral infections, associates with age-related alterations in the peripheral T-cell pool [7]. Chronic infection especially by cytomegalovirus (CMV) has a dramatic influence on the T-cell compartment, both on CD8+ and CD4+ T-cells [8,9]. CMV interferes with different aspects of immune responses, and HLA, KIR, and GM genes have been shown to play a crucial role in CMV control (for review, see [10]). Recent epidemics of emerging pathogens that have a differential immune response depending on age, such as SARS-CoV-2, illustrate the crucial importance to understand the basis of an adequate response to new antigens and the relevance of factors such as age or CMV infection.

The seroprevalence of CMV worldwide has been estimated to reach from 45 to 100% in the general population, depending on socio-economic status and age [11]. Persistent CMV infection requires continuous control by the host immune system that is itself altered with age [12,13]. Studies have revealed that CMV infection associates with an increased risk of death and cardiovascular diseases [14,15], and CMV-seropositivity was identified as one of the immune parameters of the “Immune Risk Phenotype” (IRP). The IRP was developed from the Swedish OCTO/NONA longitudinal studies, which evaluated factors predicting mortality and morbidity at very old age [16,17]. The negative impact of CMV-seropositivity is potentially associated with the accumulation of antigen-specific oligoclonally expanded CD8+CD28−CD57+ T-cells, affecting the T-cell repertoire, and the functional abilities of other T-cell populations [3,18,19]. Overall, these CD8+CD28−CD57+ T-cells have a limited proliferative capacity, and some may have lost this ability altogether as a consequence of the replicative senescence induced by repeated antigenic stimulation, whereas others can still be stimulated to up-regulate telomerase and proliferate [20]. In light of that, CMV chronic infection may be viewed as a key driver of some aspects of immunosenescence in humans with negative clinical consequences [9].

In contrast, in early life, CMV may have a beneficial impact on host immune defenses. In mice, young animals infected with murine CMV (MCMV) exhibit a better response to influenza virus than uninfected mice [21] as well as better resistance to bacterial pathogens [22]. In humans, CMV-seropositivity may associate with better immune responses toward the influenza vaccination in younger but not older individuals [21], and there is also some evidence that CMV infection improves immune responses in Gambian infants. Thus, CMV-seropositive infants exhibited a stronger CD8+ T-cell response to Staphylococcal Enterotoxin B (SEB) superantigen, and their antibody response to measles vaccination correlated with the IFN-γ response to CMV [23]. In line with these results, in our previous studies, we have demonstrated that CMV-seropositivity is associated with the expansion of highly polyfunctional CD57+ T-cells (CD4+, CD8+, and NKT-like cells) in young and middle-aged individuals in response to different stimuli [24,25,26]. Polyfunctionality has generally been considered a sign of good immune response status and is defined as the ability of one cell to simultaneously produce multiple cytokines [27]. Thus, this CMV-driven expansion of polyfunctional CD57+ T-cells supports the concept that herpesvirus latency, such as CMV, might provide cross-protection against other pathogens under certain circumstances in early life [22,28] but be detrimental in later life, which is a classic case of antagonistic pleiotropy.

In the current study, we have expanded our results to elderly individuals, as we have been able to recruit both CMV-seronegative and seropositive elderly individuals to assess for the first time the effects of age and CMV infection on T-cells, using Staphylococcal enterotoxins, such as SEB, which are the most potent known T-cell mitogens [29,30], to probe the functionality of T-cell subsets. T-cell challenge with SEB superantigen triggers massive cytokine release, up-regulation of activation markers, cytotoxicity, and proliferation [31]. Therefore, we selected this strong stimulus to study the polyfunctional responses (degranulation, IFN-γ and TNF-α production) of CD4+, CD8+, CD8+CD56+ (NKT-like), and CD4-CD8- (DN) T-cells, according to their CD57 expression, in a cohort of healthy middle-aged and older individuals stratified by CMV serostatus.

## 2. Results

### 2.1. Effect of Age and CMV Infection on T-Cell Responses to SEB Stimulation

SEB-induced T-cell responses were evaluated by multicolor flow cytometry measuring IFN-γ, TNF-α, and CD107a (degranulation) simultaneously (Figure S1). FlowJo’s Boolean analysis of IFN-γ, TNF-α, and CD107a expression generated eight different possible functional combinations per T-cell subset. However, some of these combinations were not considered biologically meaningful, given that their cell counts were very low. PI was evaluated among the four groups (Middle-aged CMV-seropositive, Middle-aged CMV-seronegative, Old CMV-seropositive, and Old CMV-seronegative) to assess the effect of age and CMV infection on T-cell responses to SEB.

### 2.2. CD4+ T-Cells

**Figure 1:**
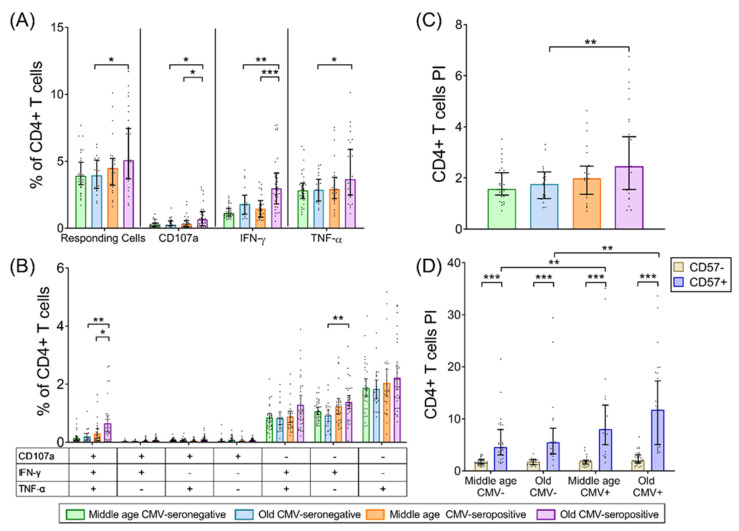
CD4+ T-cell response to SEB stimulation in healthy individuals stratified by CMV serostatus and age. (**A**) Percentage of CD4+ T-cells that have any studied response to SEB, either total degranulation (CD107a) or the production of either of the cytokines, IFN-γ or TNF-α. (**B**) Graph shows the Boolean analysis result of SEB-induced responses (CD107a, IFN-γ and/or TNF-α). Scatter graphs show the magnitude of SEB responses in each functional category expressed as percentage of CD4+ T-cells. The combination of functions studied is indicated in the table below the scatter graphs. (**C**) The polyfunctionality index (PI) of CD4+ T-cells in response to SEB. (**D**) PI of CD4+ T-cells according to CD57 expression. Horizontal black lines indicate interquartile ranges, ranging from the 25th to the 75th percentile. The median for SEB-induced CD4+ T-cell responses is indicated by the bars’ upper limit. Results were considered significant at * p < 0.05, ** p < 0.01, and *** p < 0.001.

Analysis of total SEB-responding CD4+ T-cells, considered as those capable of any kind of response (CD107a, IFN-γ, TNF-α), showed that CMV-seropositive older individuals had a higher response compared with CMV-seronegative (Figure 1A). When each function was analyzed separately, we observed a gain of all functions studied (CD107a, IFN-γ, TNF-α) in CMV-seropositive older individuals compared with CMV-seronegative (Figure 1A). Furthermore, in CMV-seropositive individuals, the percentage of CD4+ T-cells that were CD107a+ or IFN-γ+ increased with age. In contrast, no significant differences were observed with age in CMV-seronegative individuals. Finally, TNF-α did not change with age, regardless of the CMV-serostatus (Figure 1A).

Analysis of CD4+ T-cell polyfunctionality showed that CD107a was always expressed in combination with IFN-γ and TNF-α production. The percentage of these trifunctional CD4+ T-cells increased with age, but only in CMV-seropositive individuals. These cells also accumulate with CMV-seropositivity in the older group (Figure 1B). Additionally, monofunctional (IFN-γ+) CD4+ T-cells increased with CMV-seropositivity in older individuals.

Comparison of CD4+ T-cell polyfunctional indices (PI) among groups showed an increase with CMV-seropositivity in the older group but no effect of age alone (Figure 1C). Thus, our results indicate that in the absence of CMV, age has no effect on the size or polyfunctionality of the CD4+ T-cell response to SEB.

We further analyzed the expression of CD57 in the CD4+ T-cell subset. No significant differences of CD57 expression were observed between resting and SEB-stimulated CD4+ T-cells. Expansions of CD4+CD57+ T-cells were only observed in CMV-seropositive individuals regardless of age (Figure S2). In CMV-seronegative individuals, the expression of CD57 by CD4+ T-cells was absent or very low. Thus, the accumulation of CD4+CD57+ T-cells only occurs in the context of CMV infection and is independent of age.

We further analyzed the PI of CD4+ T-cells according to CD57 expression. Our results showed that the CD4+CD57+ T-cell PI was significantly higher than in CD4+CD57− T-cells in the four groups studied, despite the low CD4+CD57+ T-cell percentages found in CMV-seronegative individuals. Furthermore, when comparing the PI of CD4+CD57+ T-cells among groups, we observed an increase with CMV-seropositivity both in middle-aged and older individuals. However, no effect of age alone was found. The PI ofCD4+CD57− T-cells was not affected by CMV infection or age (Figure 1D).

### 2.3. CD8+ T-Cells

**Figure 2:**
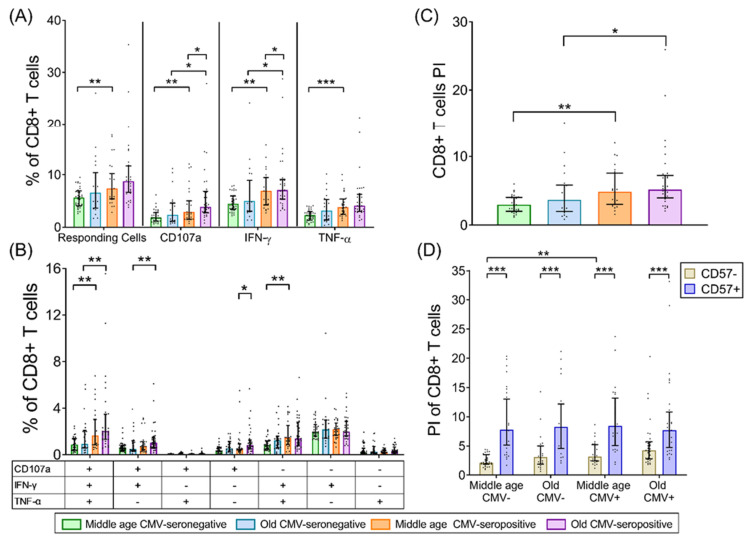
CD8+ T-cell response to SEB stimulation in healthy individuals stratified by CMV serostatus and age. (**A**) Percentage of CD8+ T-cells with any studied response to SEB, either total degranulation (CD107a) or the production of either of the cytokines, IFN-γ or TNF-α. (**B**) Graph shows the Boolean analysis result of SEB-induced responses (CD107a, IFN-γ, and/or TNF-α). Scatter graphs show the magnitude of SEB responses within each functional category, which were expressed as percentage of CD8+ T-cells. The combination of functions studied is indicated in the table below the scatter graphs. (**C**) Polyfunctionality index (PI) of CD8+ T-cells in response to SEB. (**D**) PI of CD8+ T-cells according to CD57 expression. Horizontal black lines indicate interquartile ranges, ranging from the 25th to the 75th percentile. The median for CD8+ T-cells SEB-induced responses is indicated by the bars’ upper limit. Results were considered significant at * p < 0.05, ** p < 0.01, and *** p < 0.001.

Similar to CD4+ T-cells, CD8+ T-cells total response to SEB remained unaffected by age. However, in middle-aged individuals, CMV seropositivity was associated with an increased percentage of SEB-responding CD8+ T-cells (Figure 2A). In older individuals, there was a similar trend, but this was not statistically significant. When we analyzed each function separately, we observed an increase in CD107a+ and IFN-γ+ CD8+ T-cells in CMV-seropositive individuals independently of age. These cells accumulate also with age, but only in CMV-seropositive individuals (Figure 2A). Additionally, TNF-α+ CD8+ T-cells increased with CMV-seropositivity in middle aged individuals, but again, age alone did not have any effect (Figure 2A).

Analysis of CD8+ T-cell polyfunctionality showed a significant increase in trifunctional cells with CMV-seropositivity, independent of age (Figure 2B). CMV-seropositivity was also associated with higher percentages of bifunctional IFN-γ+ TNF-α+ CD8+ T-cells in middle-aged individuals and bifunctional IFN-γ+CD107a+ CD+ T-cells in older individuals (Figure 2B). Additionally, the percentage of CD107a+ monofunctional cells increased with age but only in CMV-seropositive individuals (Figure 2B).

The analysis of the CD8+ T-cell PI showed that CMV-seropositive individuals had higher PI than CMV-seronegative in both groups studied (Figure 2C), and no effect of age per se was observed. Therefore, as in the CD4+ subset, the CD8+ T-cell response to SEB is not affected by age either with regard to size or polyfunctionality.

Analysis of CD57 expression by CD8+ T-cells showed an association with CMV-seropositivity and not with age (Figure S2). No significant differences in CD57 expression were observed between resting and SEB stimulated CD8+ T-cells. Furthermore, the CD8+CD57+ T-cell PI was significantly higher than in CD57− T-cells in all groups studied. When comparing the CD8+CD57+ T-cell PI among groups, no effect of age or CMV infection was observed (Figure 2D). Nevertheless, we did see a significant increase in the PI value of CD8+CD57− T-cells with CMV-seropositivity, but only in middle-aged individuals (Figure 2D).

### 2.4. NKT-Like (CD8+CD56+) T-Cells

**Figure 3:**
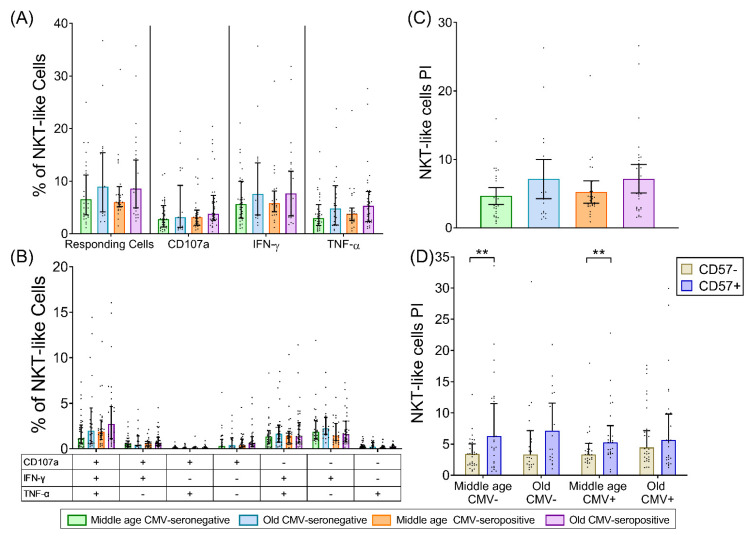
NKT-like (CD8+CD56+) T-cell response to SEB stimulation in healthy individuals stratified by CMV serostatus and age. (**A**) Percentage of NKT-like cells that have any studied response to SEB, either total degranulation (CD107a) or the production of either of the cytokines, IFN-γ or TNF-α. (**B**) Graph shows the Boolean analysis result of SEB-induced responses (CD107a, IFN-γ, and/or TNF-α). Scatter graphs show the magnitude of SEB responses in each functional category, which were expressed as percentage of NKT-like cells. The combination of functions studied is indicated in the table below the scatter graphs. (**C**) The polyfunctionality index (PI) of NKT-like cells in response to SEB. (**D**) PI of NKT-like cells according to CD57 expression. Horizontal black lines indicate interquartile ranges, ranging from the 25th to the 75th percentile. The median for NKT-like SEB-induced responses is indicated by the bars´ upper limit. Results were considered significant at ** p < 0.01.

Our results indicate that NKT-like cells are expanded in CMV-seropositive individuals in both age groups but not with age alone (Figure S3). However, NKT-like cell responses to SEB, including their PI, were not affected either by age or CMV infection (Figure 3A–C).

As in CD4+ and CD8+ subsets, CD57 expression by NKT-like cells increased with CMV infection independently of age, and age per se had no effect (Figure S2). Of note, CD57 expression was much higher in the NKT-like subset than in the other T-cell subpopulations. The PI of NKT-like CD57+ T-cells was higher than that of the CD57− counterpart but only in middle-aged individuals (Figure 3D). No significant effect of age or CMV infection was observed on the NKT-like cell PI regardless of CD57 expression.

### 2.5. CD4-CD8- T-Cells (DN T-Cells)

**Figure 4:**
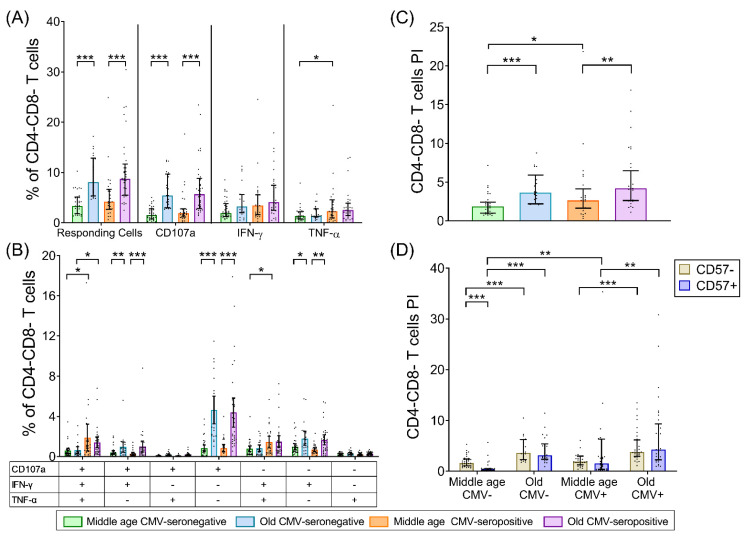
DN (CD4-CD8-)T-cell response to SEB stimulation in healthy individuals stratified by CMV serostatus and age. (**A**) Percentage of DN T-cells that have any studied response to SEB, either total degranulation (CD107a) or the production of either of the cytokines, IFN-γ or TNF-α. (**B**) Graph shows the Boolean analysis result of SEB-induced responses (CD107a, IFN-γ, and/or TNF-α). Scatter graphs show the magnitude of SEB responses within each functional category, expressed as percentage of DN T-cells. The combination of functions studied is indicated in the table below the scatter graphs. (**C**) Polyfunctionality index (PI) of DN T-cells in all groups in response to SEB. (**D**) PI of DN T-cells according to CD57 expression. Horizontal black lines indicate interquartile ranges, ranging from the 25th to the 75th percentile. The median for DN T-cells SEB-induced responses is indicated by the bars´ upper limit. Results were considered significant at * p < 0.05, ** p < 0.01, and *** p < 0.001.

In contrast to CD4+ and CD8+ subsets, the DN T-cell total SEB-response was not altered with CMV-seropositivity. However, the percentage of responding cells was higher in older individuals than in middle-aged subjects, independent of CMV-serostatus (Figure 4A). When we analyzed each function separately, we observed the same increase with age of CD107a+ DN T-cells as in other subsets. CMV-seropositivity was only associated with an increase in the percentage of TNF-α-producing DN T-cells and again only in middle-aged individuals (Figure 4A).

Analysis of DN T-cell polyfunctionality showed that CMV-seropositivity was associated with an increase in trifunctional cells, regardless of age. Additionally, in middle-aged individuals, CMV-seropositivity was also associated with an increase in bifunctional IFN-γ+TNF-α+ cells (Figure 4B). However, age was associated with an increase in bifunctional CD107a+IFN-γ+, monofunctional CD107a+, and monofunctional IFN-γ+ DN T-cells, independently of CMV serostatus. Of note, in older individuals, regardless of CMV-seropositivity, the most frequent functional category was CD107a+ monofunctional cells that are present only at very low frequencies in middle-aged individuals.

The DN T-cell PI increased with age independently of CMV infection and with CMV-seropositivity, again only in middle-aged individuals (Figure 4C). Therefore, the response to SEB of DN T-cells increased with age but not very much with CMV infection, and this was mainly due to an increase in their degranulation capacity (CD107a expression).

As in the other T-cell subsets, DN CD57+ T-cells were expanded only in CMV-seropositive individuals, and no effect of age was observed (Figure S2). No significant differences in CD57 expression were observed between resting and SEB stimulated DN T-cells. Interestingly, the DN T-cell PI did not change with CD57 expression, except in middle-aged CMV-seronegative individuals where the CD57+ cell PI was almost absent. Moreover, the PI of both CD57+ and CD57− DN T-cells increased with age independently of CMV-seropositivity (Figure 4D). The only change observed with CMV was an increase in CD57+ DN T-cells PI in middle-aged individuals. In older individuals, there was a similar trend, but this was not statistically significant, which was probably due to the increase in these cells’ PI with age.

## 3. Discussion

In humans, repetitive replication of T-cells is associated with the loss of CD28 and the acquisition of CD57. Thus, CD28− and CD57+ T-cells are considered to be late- or terminally differentiated T-cells characterized by low telomerase activity and shorter telomeres compared with CD28+CD57− T-cells. At least some of these CD28-CD57+ T-cells may be senescent [32,33]. It has been shown that besides age, persistent CMV infection is also associated with the accumulation of these highly differentiated T-cells [34,35]. Accumulating evidence supports a detrimental role of senescent T-cells in several chronic inflammatory clinical conditions, including cardiovascular diseases such as atherosclerosis and myocardial infarction (for a review, see [36,37]).

Our results show that in middle-aged and older overtly healthy individuals, the main factor driving the expansion of CD57+ T-cells is CMV infection. However, from the fourth decade onwards, these cells do not accumulate further with age. In previous work, we showed that the percentage of CD8+CD57+ T-cells was similar between young and middle-aged CMV-seropositive individuals [25]. Therefore, here, we extend our previous findings [24,25,26,38] to show that CD57 expression by T-cells is not only a hallmark of CMV infection in young individuals but also at older ages. Accordingly, once CMV infection takes place, CD57+ T-cells will expand, and after that, their percentage will remain rather stable over time. Thus, our results argue against the consensus that the expansion of these cells is a sign of chronological aging.

Regarding CD57+ T-cell functional capacities, our data also indicate that CD57+ T-cells are more polyfunctional than CD57− T-cells at any age, with one exception, namely the DN T-cell subset, where the PI does not change with CD57 expression. Nonetheless, we did observe an increase in trifunctional DN T-cells with CMV seropositivity, and the total PI of DN T-cells increased with CMV infection in middle-aged individuals. In this subset, CD57+ T-cells also accumulate in CMV-seropositive individuals.

Our results regarding CD4+CD57+ T-cell expansions with CMV infection are also in agreement with the observation that CMV, but not aging, has a significant effect on the expansion of pro-atherogenic CD4+CD28− T-cells [39]. These cells (that also express CD57) are cytotoxic, capable of causing vascular damage, and their expansion is associated with autoimmune and cardiovascular disease (for a review, see [36,37]). Here, we show that similar to young individuals [24], at older ages, CD4+CD57+ T-cells are also more polyfunctional (CD107a, IFN-γ, and TNF-α) than their CD57− counterparts. Additionally, the percentage of these polyfunctional cells correlated with the percentage of total CD4+CD107a+ (i.e., cytotoxic) T-cells (Spearman correlation *p*-value = 0.03, Figure S4). Notably, CMV-seronegative individuals had very low or no percentages of both cytotoxic CD4+ T-cells (CD1017a+) and CD4+CD57+ T-cells, supporting the conclusion that the expansion of cytotoxic CD4+ T-cells only occurs with CMV infection.

Higher frequencies of CD4+CD57+ T-cells have been associated with poorer prognosis in several diseases. In acute heart failure patients, high percentages of these cells are associated with the development of cardiovascular events (defined as heart failure-associated mortality, transplantation, or rehospitalization) [40]. In end-stage renal disease patients, the frequency of CD4+CD57+ T-cells is associated with atherosclerotic changes [41], and in multiple sclerosis, their frequency is associated with disease severity and poorer prognosis [42]. In addition, in acute heart failure patients, percentages of IFN-γ+ and TNF-α+cells are higher within the CD4+CD57+ than the CD57− T-cell subset, and CD4+CD57+IFN-γ+T-cells were increased in patients compared with healthy individuals in response to anti-CD3 stimulation [40]. In our hands, the PI of CD4+ T-cells overall and the CD4+CD57+ T-cell fraction increased with CMV-seropositivity, but not age, supporting a significant role of CMV in the development of cardiovascular disease that can be explained, at least in part, through the expansion of these proinflammatory and cytotoxic CD4+CD57+ T-cells. Consistent with this, a link between CMV infection, CD4+CD28− T-cell expansions, and autoimmune and cardiovascular disorders has been previously suggested [36,43].

Our data may be of some practical clinical importance because CMV infection can be treated, and the expansion of these cytotoxic proinflammatory cells could potentially be prevented. In this respect, the use of Valacyclovir as anti-CMV treatment in patients with Antineutrophil Cytoplasmic Antibody (ANCA)-Associated Vasculitis was shown not only to suppress CMV reactivation but was also associated with a reduction of the CD4+CD28− T-cell frequency. Moreover, in a different context, a lower frequency of these cells correlated with improved responses to pneumococcal vaccination [44]. These results strongly suggest that the CMV-driven expansion of CD4+CD28−T-cells, and by extension CD4+CD57+ T-cells, might have a detrimental effect on the immune response to vaccination. These results, together with the proof of principle of the potential benefit of using anti-CMV treatments in ANCA-Associated Vasculitis, support the possible application of anti-CMV therapy in any clinical situation where CD4+CD57+ T-cells are implicated, including impaired responses to infection and vaccination.

Our results show that CD8+CD57+ T-cells are also expanded in CMV-seropositive individuals, both middle-aged and older. As in CD4+ T-cells, the expression of CD57 was associated with higher polyfunctionality determined by the PI value, this increase in the polyfunctionality of CD4+ and CD8+ T-cells being a hallmark of CMV infection. Indeed, it has been shown that CMV-specific CD8+ T-cells that produce IFN-γ and TNF-α as well as express CD107a play an important role in controlling this viral infection in the context of allogeneic stem cell transplantation [45]. Similarly, CMV-specific CD107a+IFN-γ+CD8+ T-cells are important for controlling CMV infection in rhesus macaques [46].

The expansion of CD8+CD57+ T lymphocytes is associated with CMV viremia in solid organ transplantation. These cells were characterized by IFN-γ and granzyme B production, and their expansion was associated with CMV-specific immune responses in pediatric cardiac transplantation [47]. Moreover, in renal transplant recipients, CMV induced the expansion of highly functional memory CD57+ T-cells [48], and during active CMV replication, highly differentiated CD8+CD57+ T-cells acquire cytotoxic activity [48]. Additionally, the frequency of total CD8+CD28− T-cells has been related to the specific immune response to CMV one year after solid organ transplantation [49].

As has been pointed out before, the effects of CMV infection and age differ among T-cell subsets. As reported here, CD4+ and CD8+ T-cells display a functional shift associated with CMV seropositivity, while NKT-like and DN T-cell subsets do not. Specifically, NKT-like cell functionality was not affected either by age or CMV, while DN T-cells’ response to SEB and PI was mainly altered by age. In individuals over 65 years of age, the impact of CMV infection on DN T-cell functionality seems to be diluted by the effect of aging.

The increased PI of DN T-cells with age seems to be due to a gain in cytotoxicity (CD107a marker of degranulation) rather than to higher cytokine production. DN T-cells are mainly gamma-delta T lymphocytes, and studies on the effect of its aging have yielded disparate results [50,51,52,53]. CMV does not stimulate Vdelta2+ cells, but it does stimulate Vdelta2− T-cells. These are characterized by the accumulation of highly differentiated Vδ2− subsets with time, in contrast to Vδ2+ T-cells, which are decreased in old individuals independently of CMV serostatus and maintain a less differentiated phenotype [50]. Although a limitation of our study is that we have not characterized the different gamma-delta subsets within the DN T-cells, our results clearly show a higher capacity of degranulation of these cells in older adults, independent of CMV serostatus.

It has been reported that NKT-like cells expand with age and in certain disease states. Our previous work [26,38] showed that in CMV-seropositive individuals of up to 60 years of age, there was no effect of age on the expansion of these cells. We only observed an increase in NKT-like cell percentages when comparing young CMV-seronegative individuals and middle-aged CMV-seropositive individuals, indicating that CMV is an important factor for their expansion. However, there was no increase in NKT-like cell frequency with CMV in young individuals. To shed light on whether their accumulation is due to CMV infection alone or a combined effect with chronological aging, we analyzed the percentage of this cell subset at older age. Our results demonstrate that NKT-like cells expand in CMV-seropositive individuals over the age of 40 and onwards, and that age per se had no effect on their accumulation. Furthermore, we have also observed a significant expansion of NKT-like CD57+ cells in CMV-seropositive individuals, which is unrelated to aging. Earlier reports have shown heterogeneous results regarding the possible effect of aging on the number and function of NKT-like cells [54]. In the present study, we show that NKT-like cells accumulate from middle age onwards with CMV infection but not age alone, although no functional alterations were observed in this subset. These results are in contrast to other studies showing a higher functionality of CD56+ T-cells (determined by levels of CD107a and proinflammatory cytokines) in CMV-seropositive individuals [55]. These differences could be due to the fact that the age of the young group ranged from 23 to 60 years old in that study.

CMV infection induces the expansion of CD57+ T-cells, but whether this has a beneficial or detrimental or neutral role for immunity and long-term effects on health and its direct effect on response to pathogens and vaccines is still under debate. Nevertheless, there is mounting evidence that CMV-seropositivity is associated with a reduced response to both invasion with a novel pathogen and to vaccination Some studies have reported a beneficial effect of CMV infection in both young and older adults in response to vaccination [21,23], while other studies suggest that it can be detrimental [12,13]. The increased functionality of CD57+CD4+ and CD57+CD8+ T-cells shown can be considered beneficial, but under certain pathological conditions, CD57+ T-cells have immunosuppressive activity [56,57]. Thus, it is essential to further investigate the effects of CMV on the immune response at older ages. In this regard, our present study complements our previous investigations allowing us to dissect the effect of age and CMV in older individuals. This is especially important for vaccine development, as it has been shown for the influenza vaccines Fluad^®^ or Intanza^®^, especially designed for elderly individuals [58,59]. A poor response to Intanza^®^ was associated with CMV seropositivity, but only in older individuals [58,60]. In addition, it has been shown that older CMV-seropositive individuals have lower frequencies of influenza specific memory T-cells than CMV-seronegative, but this was not observed in younger individuals [61]. However, despite the lower frequencies of influenza-specific T-cells found in CMV-seropositive older individuals, they exhibited a significantly higher IFNγ T-cell response to influenza virus in the acute phase of the disease compared to CMV-seronegative older individuals [61]. These results agree with our results showing an increased polyfunctionality of CD4 and CD8 T-cells with CMV-seropositivity in older donors.

Our results regarding CD57+ T-cells expansion in CMV-seropositive older donors are also of interest in the study of CMV infection in transplant recipients where CMV infection represents a significant complication. CD8+CD57+ TEMRA cells increased over time after transplant specifically in CMV-seropositive but not CMV-seronegative recipients, and changes in CD8+ T-cells compatible with accelerated immune aging have been observed in CMV-seropositive transplant recipients [62]. Our results showing that CD57+ T-cells are mainly expanded in CMV-seropositive individuals, and maintain cytokine production and polyfunctionality independently of age, suggest that CD57+ T-cells may contribute to the promotion of adverse inflammatory outcomes, such as late graft dysfunction, chronic kidney rejection, cancer, and atherosclerosis observed after kidney transplant associated with CMV infection.

The results presented also highlight the relevance of considering the heterogeneity of CMV seropositivity and aging in the design of clinical trials or new vaccines in some clinical conditions such in HIV patients. Thus, CMV-induced chronic immune activation and premature immunosenescence in people living with HIV must be taken into consideration not only when comparing trial outcomes between various populations exhibiting diverse CMV positivity rates but also for HIV vaccine development [63].

Therefore, immunological treatments should consider both age and CMV infection as a major factor. This strengthens the need for validation studies with not only the aim to present something novel but also to confirm findings in different populations.

Our results support the view that CMV is a major driving force for the expansion of CD57+ T-cells, and that these cells are more polyfunctional than their CD57-negative counterparts within the CD4+ and CD8+ subsets (including NKT-like cells). Age has no significant effect on either the frequencies of CD57+ T-cells or their polyfunctionality when only CMV seronegative individuals are considered. Thus, our results indicate that the CD57+ T-cell population might play an important role for antiviral control, which is in line with their high cytotoxic and polyfunctional activity. The association of these T-cell expansions with CMV infection and disease underlines the necessity of considering CMV serology in any study regarding immunosenescence and emphasizes that the price of immune protection is always some degree of immunopathology.

## 4. Materials and Methods

### 4.1. Subjects

A total of 119 individuals from the Leiden Longevity Study (LLS) cohort was included in the present study. The Medical Ethics Committee of Leiden University Medical Center approved the study (Biomarkers of the rate of ageing CME protocol P06.059), and informed consent was obtained from all subjects. Details of the LLS study have been published previously [64,65,66]. Of these individuals, 62 were middle-aged and 57 were older individuals. Subjects were stratified according to CMV serology (CMV-seropositive and CMV-seronegative) (Table 1). Differences in the F/M ratio were a result of convenient sampling.

### 4.2. CMV Serology

Details of CMV serology of the LLS study have been published previously [64,65,66].

### 4.3. Stimulation, Intracellular Staining, and Detection of CD107a Expression

Cryopreserved PBMCs were thawed, cells were washed and resuspended in X-Vivo 15 Medium (Lonza, Cologne, Germany) and placed in a 96-well U-bottom plate at 2 × 10^6^ cells/mL concentration (250 µL final volume). They were allowed to rest for 1 h at 37 °C in a standard incubator (humidified CO_2_ atmosphere). Following this, costimulatory antibodies (anti-CD28 and anti-CD49d; 1 µL/mL each; BD Biosciences, Franklin Lakes, NJ, USA) and anti-CD107a-APC-H7 (BD Biosciences), for degranulation detection [67], were added to all wells. Staphylococcal Enterotoxin B superantigen (SEB, Sigma-Aldrich, Burlington, MA, USA) was added at a final concentration of 1 µg/mL to the corresponding wells. For each individual, a negative control containing only anti-CD28 and anti-CD49d was included to measure antigen-independent stimulation. The plate was placed in a standard incubator (37 °C, humidified CO_2_ atmosphere) and, after 1 h, each well received the addition of monesin (Golgistop, 0.67 µL/mL; BD Biosciences) and brefeldin A (Golgi Plug 1 µL/mL; BD Biosciences). Then, cells were incubated for an additional 4 h.

Following incubation, cells were washed twice with PFEA buffer (PBS, 2% FCS, 2 mM EDTA, and 0.01% sodium azide) and were treated with human Ig, GAMUNEX (Bayer, Leverkusen, Germany), and ethidium monoazide bromide (EMA) (Invitrogen, Karlsruhe, Germany) for 10 min on ice to block FcRs and label nonviable cells. Then, cells were stained with antibodies to surface molecules (CD57-Pacific Blue, Biolegend, and CD56-brilliant violet 605, BD Biosciences) and were incubated for 20 min at 4 °C in the dark. Then, cells were washed, fixed, and permeabilized with Cytofix/Cytoperm solution according to the manufacturer’s instructions (BD Biosciences) and stained intracellularly with CD3-APC, CD4-Briliant Violet 711 (Biolegend, San Diego, CA, USA), CD8-PerCP, IFN-γ-PE-Cy7 (BD Biosciences), and TNF-α-Alexa Fluor 700 (e-Biosciences, Waltham, MA, USA) antibodies. All antibodies were titrated before use. Stained cells were analyzed by flow cytometry the following day, and Comp Beads BD were used prior to measurement as a reference control to compensate for the potential LSRII performance variation between samples.

### 4.4. Flow Cytometry and Data Analysis

Flow cytometric analysis was performed on an LSR II cytometer with FACSDiva software (BD Biosciences). The spectral overlap between all channels was calculated automatically by the BD FACSDiva software after measuring negative and single-color controls. Data were analyzed using FlowJo v10 software (Tree Star, Portland, OR, USA).

For data analysis, the first gate was time gate vs. side scatter (SSC-A) to detect differences in the flow; then, lymphocytes were gated in a forward scatter (FSC-A) vs. side scatter (SSC-A) according to their size and granularity. After singlet gating, the EMA-negative population was selected to exclude dead cells in an EMA vs. CD3 plot, and EMA-CD3+ cells were characterized as living T-cells. T-cell subsets including CD4+, CD8+, CD8+CD56+ (NKT-like), and CD4-CD8- (DN) were gated subsequently as illustrated in (Figure S1A). The average number of events acquired for each subset was 151,049 cells for CD4+ subset, 53,292 cells for CD8+, 3583 cells for NKT-like, and 13,588 cells for DN. Within each T-cell subset, individual gates were made for the detection of CD57 expression and to identify positive responses (CD107a, IFN-γ, TNF-α) (Figure S1B). Boolean gating was performed to create a full array of possible combinations of response patterns from the T-cell subsets or from each of the CD57+/- subpopulations; however, some of the possible combinations of the Boolean analysis of IFN-γ, TNF-α, and CD107a expression were not considered biologically meaningful, given that their cell counts were very low. Positive responses were reported after background subtraction. Gates for CD107a, IFN-γ, and TNF-α were set based on the unstimulated control. Fluorescence minus one (FMO) control was used for phenotype gates.

The polyfunctional index (PI) was calculated as described by Larsen et al. (Funky Cells Toolbox; http://www.funkycells.com/main/) [68]. The PI enables comparative and correlative parametric and non-parametric statistical tests by numerically evaluating the degree and variation of polyfunctionality.

### 4.5. Statistical Analysis

For statistical analysis, to test normality, a Shapiro–Wilk test was performed. No normality was found. According to this, a Kruskal–Wallis *H* test (non-parametric test) with correction for multiple comparisons was used for direct comparison of the four groups. Then, those variables in which we found a statistical significant difference were analyzed using the Mann–Whitney *U* non-parametric test for comparing data among the specific sample pairs. All graphs reflect only the Mann–Whitney derived *p*-values, and graphs were done using GraphPad Prism (version 8.1). For comparison between CD4+CD107a+ T-cells and CD4+CD57+ T-cells expression, a Spearman correlation test was used. All statistical analyses were performed with GraphPad Prism (version 8.1). *p*-values < 0.05 were considered significant.

## Figures and Tables

**Table 1:** Demographics of studied individuals (*n* = 119).

CMV	Age (Mean ± SD)	Sex (Male/Female)	No.	Group Name
Negative	45–64 (60 ± 3)	13/21	34	Middle-aged CMV-seronegative
Positive	46–63 (60 ± 3)	10/18	28	Middle-aged CMV-seropositive
Negative	65–78 (69 ± 3)	16/8	24	Older CMV-seronegative
Positive	65–73 (68 ± 2)	18/15	33	Older CMV-seropositive

## Data Availability

The data presented in this study are available on request from the corresponding author.

## Supplementary Materials

The following are available online at https://www.mdpi.com/article/10.3390/ijms22189973/s1.

## References

[1] Bonilla F.A., Oettgen H.C. (2010). Adaptive immunity. J. Allergy Clin. Immunol..

[2] Farber D.L., Yudanin N.A., Restifo N.P. (2014). Human memory T cells: Generation, compartmentalization and homeostasis. Nat. Rev. Immunol..

[3] Pawelec G., Derhovanessian E. (2011). Role of CMV in immune senescence. Virus Res..

[4] Pawelec G., Derhovanessian E., Larbi A., Strindhall J., Wikby A. (2009). Cytomegalovirus and human immunosenescence. Rev. Med. Virol..

[5] Miller R.A. (1996). The aging immune system: Primer and prospectus. Science.

[6] Cambier J. (2005). Immunosenescence: A problem of lymphopoiesis, homeostasis, microenvironment, and signaling. Immunol. Rev..

[7] Wertheimer A.M., Bennett M.S., Park B., Uhrlaub J.L., Martinez C., Pulko V., Currier N.L., Nikolich-Žugich D., Kaye J., Nikolich-Žugich J. (2014). Aging and cytomegalovirus infection differentially and jointly affect distinct circulating T cell subsets in humans. J. Immunol..

[8] Koch S., Solana R., Dela Rosa O., Pawelec G. (2006). Human cytomegalovirus infection and T cell immunosenescence: A mini review. Mech. Ageing Dev..

[9] Koch S., Larbi A., Ozcelik D., Solana R., Gouttefangeas C., Attig S., Wikby A., Strindhall J., Franceschi C., Pawelec G. (2007). Cytomegalovirus infection: A driving force in human T cell immunosenescence. Ann. N. Y. Acad. Sci..

[10] Aiello A., Accardi G., Candore G., Caruso C., Colomba C., Di Bona D., Duro G., Gambino C.M., Ligotti M.E., Pandey J.P. (2019). Role of Immunogenetics in the Outcome of HCMV Infection: Implications for Ageing. Int. J. Mol. Sci..

[11] Cannon M.J., Schmid D.S., Hyde T.B. (2010). Review of cytomegalovirus seroprevalence and demographic characteristics associated with infection. Rev. Med. Virol..

[12] Khan N., Shariff N., Cobbold M., Bruton R., Ainsworth J.A., Sinclair A.J., Nayak L., Moss P.A. (2002). Cytomegalovirus seropositivity drives the CD8 T cell repertoire toward greater clonality in healthy elderly individuals. J. Immunol..

[13] Ouyang Q., Wagner W.M., Zheng W., Wikby A., Remarque E.J., Pawelec G. (2004). Dysfunctional CMV-specific CD8(+) T cells accumulate in the elderly. Exp. Gerontol..

[14] Pawelec G., McElhaney J.E., Aiello A.E., Derhovanessian E. (2012). The impact of CMV infection on survival in older humans. Curr. Opin. Immunol..

[15] Simanek A.M., Dowd J.B., Pawelec G., Melzer D., Dutta A., Aiello A.E. (2011). Seropositivity to cytomegalovirus, inflammation, all-cause and cardiovascular disease-related mortality in the United States. PLoS ONE.

[16] Olsson J., Wikby A., Johansson B., Lofgren S., Nilsson B.O., Ferguson F.G. (2000). Age-related change in peripheral blood T-lymphocyte subpopulations and cytomegalovirus infection in the very old: The Swedish longitudinal OCTO immune study. Mech. Ageing Dev..

[17] Ferrando-Martinez S., Romero-Sanchez M.C., Solana R., Delgado J., de la Rosa R., Munoz-Fernandez M.A., Ruiz-Mateos E., Leal M. (2013). Thymic function failure and C-reactive protein levels are independent predictors of all-cause mortality in healthy elderly humans. Age.

[18] Weng N.P., Akbar A.N., Goronzy J. (2009). CD28(-) T cells: Their role in the age-associated decline of immune function. Trends Immunol..

[19] Chou J.P., Effros R.B. (2013). T cell replicative senescence in human aging. Curr. Pharm. Des..

[20] Strioga M., Pasukoniene V., Characiejus D. (2011). CD8+ CD28- and CD8+ CD57+ T cells and their role in health and disease. Immunology.

[21] Furman D., Jojic V., Sharma S., Shen-Orr S.S., Angel C.J., Onengut-Gumuscu S., Kidd B.A., Maecker H.T., Concannon P., Dekker C.L. (2015). Cytomegalovirus infection enhances the immune response to influenza. Sci. Transl. Med..

[22] Barton E.S., White D.W., Cathelyn J.S., Brett-McClellan K.A., Engle M., Diamond M.S., Miller V.L., Virgin H.W.t. (2007). Herpesvirus latency confers symbiotic protection from bacterial infection. Nature.

[23] Miles D.J., Sanneh M., Holder B., Crozier S., Nyamweya S., Touray E.S., Palmero M.S., Zaman S.M., Rowland-Jones S., van der Sande M. (2008). Cytomegalovirus infection induces T-cell differentiation without impairing antigen-specific responses in Gambian infants. Immunology.

[24] Pera A., Vasudev A., Tan C., Kared H., Solana R., Larbi A. (2017). CMV induces expansion of highly polyfunctional CD4+ T cell subset coexpressing CD57 and CD154. J. Leukoc. Biol..

[25] Pera A., Campos C., Corona A., Sanchez-Correa B., Tarazona R., Larbi A., Solana R. (2014). CMV latent infection improves CD8+ T response to SEB due to expansion of polyfunctional CD57+ cells in young individuals. PLoS ONE.

[26] Hassouneh F., Campos C., López-Sejas N., Alonso C., Tarazona R., Solana R., Pera A. (2016). Effect of age and latent CMV infection on CD8+ CD56+ T cells (NKT-like) frequency and functionality. Mech. Ageing Dev..

[27] Boyd A., Almeida J.R., Darrah P.A., Sauce D., Seder R.A., Appay V., Gorochov G., Larsen M. (2015). Pathogen-Specific T Cell Polyfunctionality Is a Correlate of T Cell Efficacy and Immune Protection. PLoS ONE.

[28] White D.W., Suzanne Beard R., Barton E.S. (2012). Immune modulation during latent herpesvirus infection. Immunol. Rev..

[29] Fleischer B. (1994). Superantigens. APMIS.

[30] Fleischer B., Schrezenmeier H. (1988). T cell stimulation by staphylococcal enterotoxins. Clonally variable response and requirement for major histocompatibility complex class II molecules on accessory or target cells. J. Exp. Med..

[31] Whitfield S.J.C., Taylor C., Risdall J.E., Griffiths G.D., Jones J.T.A., Williamson E.D., Rijpkema S., Saraiva L., Vessillier S., Green A.C. (2017). Interference of the T Cell and Antigen-Presenting Cell Costimulatory Pathway Using CTLA4-Ig (Abatacept) Prevents Staphylococcal Enterotoxin B Pathology. J. Immunol..

[32] Appay V., van Lier R.A., Sallusto F., Roederer M. (2008). Phenotype and function of human T lymphocyte subsets: Consensus and issues. Cytom. A.

[33] Koch S., Larbi A., Derhovanessian E., Ozcelik D., Naumova E., Pawelec G. (2008). Multiparameter flow cytometric analysis of CD4 and CD8 T cell subsets in young and old people. Immun. Ageing.

[34] Elwenspoek M.M.C., Sias K., Hengesch X., Schaan V.K., Leenen F.A.D., Adams P., Meriaux S.B., Schmitz S., Bonnemberger F., Ewen A. (2017). T Cell Immunosenescence after Early Life Adversity: Association with Cytomegalovirus Infection. Front. Immunol..

[35] Focosi D., Bestagno M., Burrone O., Petrini M. (2010). CD57+ T lymphocytes and functional immune deficiency. J. Leukoc. Biol..

[36] Broadley I., Pera A., Morrow G., Davies K.A., Kern F. (2017). Expansions of Cytotoxic CD4. Front. Immunol..

[37] Pera A., Broadley I., Davies K.A., Kern F. (2017). Cytomegalovirus as a Driver of Excess Cardiovascular Mortality in Rheumatoid Arthritis: A Red Herring or a Smoking Gun?. Circ. Res..

[38] Hassouneh F., Lopez-Sejas N., Campos C., Sanchez-Correa B., Tarazona R., Solana R., Pera A. (2017). Differential Effect of Cytomegalovirus Infection with Age on the Expression of CD57, CD300a, and CD161 on T-Cell Subpopulations. Front. Immunol..

[39] Pera A., Caserta S., Albanese F., Blowers P., Morrow G., Terrazzini N., Smith H.E., Rajkumar C., Reus B., Msonda J.R. (2018). CD28. Theranostics.

[40] Youn J.C., Jung M.K., Yu H.T., Kwon J.S., Kwak J.E., Park S.H., Kim I.C., Park M.S., Lee S.K., Choi S.W. (2019). Increased frequency of CD4. Sci. Rep..

[41] Okba A.M., Abd El Raouf Raafat M., Nazmy Farres M., Abd El Nour Melek N., Amin M.M., Gendy N.N. (2019). Expanded peripheral CD4. Hum. Immunol..

[42] Peeters L.M., Vanheusden M., Somers V., Van Wijmeersch B., Stinissen P., Broux B., Hellings N. (2017). Cytotoxic CD4+ T Cells Drive Multiple Sclerosis Progression. Front. Immunol..

[43] Bano A., Pera A., Almoukayed A., Clarke T.H.S., Kirmani S., Davies K.A., Kern F. (2019). CD28. F1000Research.

[44] Chanouzas D., Sagmeister M., Faustini S., Nightingale P., Richter A., Ferro C.J., Morgan M.D., Moss P., Harper L. (2019). Subclinical Reactivation of Cytomegalovirus Drives CD4+CD28null T-Cell Expansion and Impaired Immune Response to Pneumococcal Vaccination in Antineutrophil Cytoplasmic Antibody-Associated Vasculitis. J. Infect. Dis..

[45] Muñoz-Cobo B., Solano C., Benet I., Costa E., Remigia M.J., de la Cámara R., Nieto J., López J., Amat P., Garcia-Noblejas A. (2012). Functional profile of cytomegalovirus (CMV)-specific CD8+ T cells and kinetics of NKG2C+ NK cells associated with the resolution of CMV DNAemia in allogeneic stem cell transplant recipients. J. Med. Virol..

[46] Chan K.S., Kaur A. (2007). Flow cytometric detection of degranulation reveals phenotypic heterogeneity of degranulating CMV-specific CD8+ T lymphocytes in rhesus macaques. J. Immunol. Methods.

[47] Jacobsen M.C., Manunta M.D.I., Pincott E.S., Fenton M., Simpson G.L., Klein N.J., Burch M. (2018). Specific Immunity to Cytomegalovirus in Pediatric Cardiac Transplantation. Transplantation.

[48] Makwana N., Foley B., Fernandez S., Lee S., Irish A., Pircher H., Price P. (2017). CMV drives the expansion of highly functional memory T cells expressing NK-cell receptors in renal transplant recipients. Eur. J. Immunol..

[49] Cantisán S., Páez-Vega A., Santos F., Rodríguez-Benot A., Aguado R., Rivero A., Montejo M., Torre-Cisneros J., Solana R., (REIPI) S.N.f.R.i.I.D. (2017). Impact of age and cytomegalovirus on CD8. Exp. Gerontol..

[50] Roux A., Mourin G., Larsen M., Fastenackels S., Urrutia A., Gorochov G., Autran B., Donner C., Sidi D., Sibony-Prat J. (2013). Differential impact of age and cytomegalovirus infection on the γδ T cell compartment. J. Immunol..

[51] Vasudev A., Ying C.T., Ayyadhury S., Puan K.J., Andiappan A.K., Nyunt M.S., Shadan N.B., Mustafa S., Low I., Rotzschke O. (2014). γ/δ T cell subsets in human aging using the classical α/β T cell model. J. Leukoc. Biol..

[52] Tan C.T., Wistuba-Hamprecht K., Xu W., Nyunt M.S., Vasudev A., Lee B.T., Pawelec G., Puan K.J., Rotzschke O., Ng T.P. (2016). Vδ2+ and α/ß T cells show divergent trajectories during human aging. Oncotarget.

[53] van der Geest K.S.M., Kroesen B.J., Horst G., Abdulahad W.H., Brouwer E., Boots A.M.H. (2018). Impact of Aging on the Frequency, Phenotype, and Function of CD161-Expressing T Cells. Front. Immunol..

[54] Mocchegiani E., Giacconi R., Cipriano C., Malavolta M. (2009). NK and NKT cells in aging and longevity: Role of zinc and metallothioneins. J. Clin. Immunol..

[55] Almehmadi M., Flanagan B.F., Khan N., Alomar S., Christmas S.E. (2014). Increased numbers and functional activity of CD56⁺ T cells in healthy cytomegalovirus positive subjects. Immunology.

[56] Sadat-Sowti B., Debre P., Mollet L., Quint L., Hadida F., Leblond V., Bismuth G., Autran B. (1994). An inhibitor of cytotoxic functions produced by CD8+CD57+ T lymphocytes from patients suffering from AIDS and immunosuppressed bone marrow recipients. Eur. J. Immunol..

[57] Frassanito M.A., Silvestris F., Cafforio P., Dammacco F. (1998). CD8+/CD57 cells and apoptosis suppress T-cell functions in multiple myeloma. Br. J. Haematol..

[58] Derhovanessian E., Theeten H., Hähnel K., Van Damme P., Cools N., Pawelec G. (2013). Cytomegalovirus-associated accumulation of late-differentiated CD4 T-cells correlates with poor humoral response to influenza vaccination. Vaccine.

[59] Goldeck D., Theeten H., Hassouneh F., Oettinger L., Wistuba-Hamprecht K., Cools N., Tsitsilonis O.E., Pawelec G. (2017). Frequencies of peripheral immune cells in older adults following seasonal influenza vaccination with an adjuvanted vaccine. Vaccine.

[60] Derhovanessian E., Maier A.B., Hähnel K., McElhaney J.E., Slagboom E.P., Pawelec G. (2014). Latent infection with cytomegalovirus is associated with poor memory CD4 responses to influenza A core proteins in the elderly. J. Immunol..

[61] van den Berg S.P.H., Lanfermeijer J., Jacobi R.H.J., Hendriks M., Vos M., van Schuijlenburg R., Nanlohy N.M., Borghans J.A.M., van Beek J., van Baarle D. (2021). Latent CMV Infection Is Associated with Lower Influenza Virus-Specific Memory T-Cell Frequencies, but Not With an Impaired T-Cell Response to Acute Influenza Virus Infection. Front. Immunol..

[62] Higdon L.E., Gustafson C.E., Ji X., Sahoo M.K., Pinsky B.A., Margulies K.B., Maecker H.T., Goronzy J., Maltzman J.S. (2021). Association of Premature Immune Aging and Cytomegalovirus After Solid Organ Transplant. Front. Immunol..

[63] Royston L., Isnard S., Lin J., Routy J.P. (2021). Cytomegalovirus as an Uninvited Guest in the Response to Vaccines in People Living with HIV. Viruses.

[64] Westendorp R.G., van Heemst D., Rozing M.P., Frolich M., Mooijaart S.P., Blauw G.J., Beekman M., Heijmans B.T., de Craen A.J., Slagboom P.E. (2009). Nonagenarian siblings and their offspring display lower risk of mortality and morbidity than sporadic nonagenarians: The Leiden Longevity Study. J. Am. Geriatr. Soc..

[65] Schoenmaker M., de Craen A.J., de Meijer P.H., Beekman M., Blauw G.J., Slagboom P.E., Westendorp R.G. (2006). Evidence of genetic enrichment for exceptional survival using a family approach: The Leiden Longevity Study. Eur. J. Hum. Genet..

[66] Mortensen L.H., Maier A.B., Slagbom P.E., Pawelec G., Derhovanessian E., Petersen I., Jahn G., Westendorp R.G., Christensen K. (2012). Early-life environment influencing susceptibility to cytomegalovirus infection: Evidence from the Leiden Longevity Study and the Longitudinal Study of Aging Danish Twins. Epidemiol. Infect..

[67] Lorenzo-Herrero S., Sordo-Bahamonde C., Gonzalez S., López-Soto A. (2019). CD107a Degranulation Assay to Evaluate Immune Cell Antitumor Activity. Methods Mol. Biol..

[68] Larsen M., Sauce D., Arnaud L., Fastenackels S., Appay V., Gorochov G. (2012). Evaluating cellular polyfunctionality with a novel polyfunctionality index. PLoS ONE.

