# Supplementary material for: Functional Changes of T-Cell Subsets with Age and CMV Infection
Source: Int J Mol Sci. 2021 Sep 15;22(18):9973. doi: 10.3390/ijms22189973 (PMC8465008; doi:10.3390/ijms22189973)
Supplement: Supplementary file 1 [file ijms-22-09973-s001.zip › ijms-1328844-supplementary.pdf]

International Journal of *Molecular Sciences*

MDPI

Article

Functional changes of T-cell subsets with age and CMV infection

Fakhri Hassouneh, David Goldeck, Alejandra Pera, P Eline Slagboom, Graham Pawelec and Rafael Solana

Supplementary Materials:

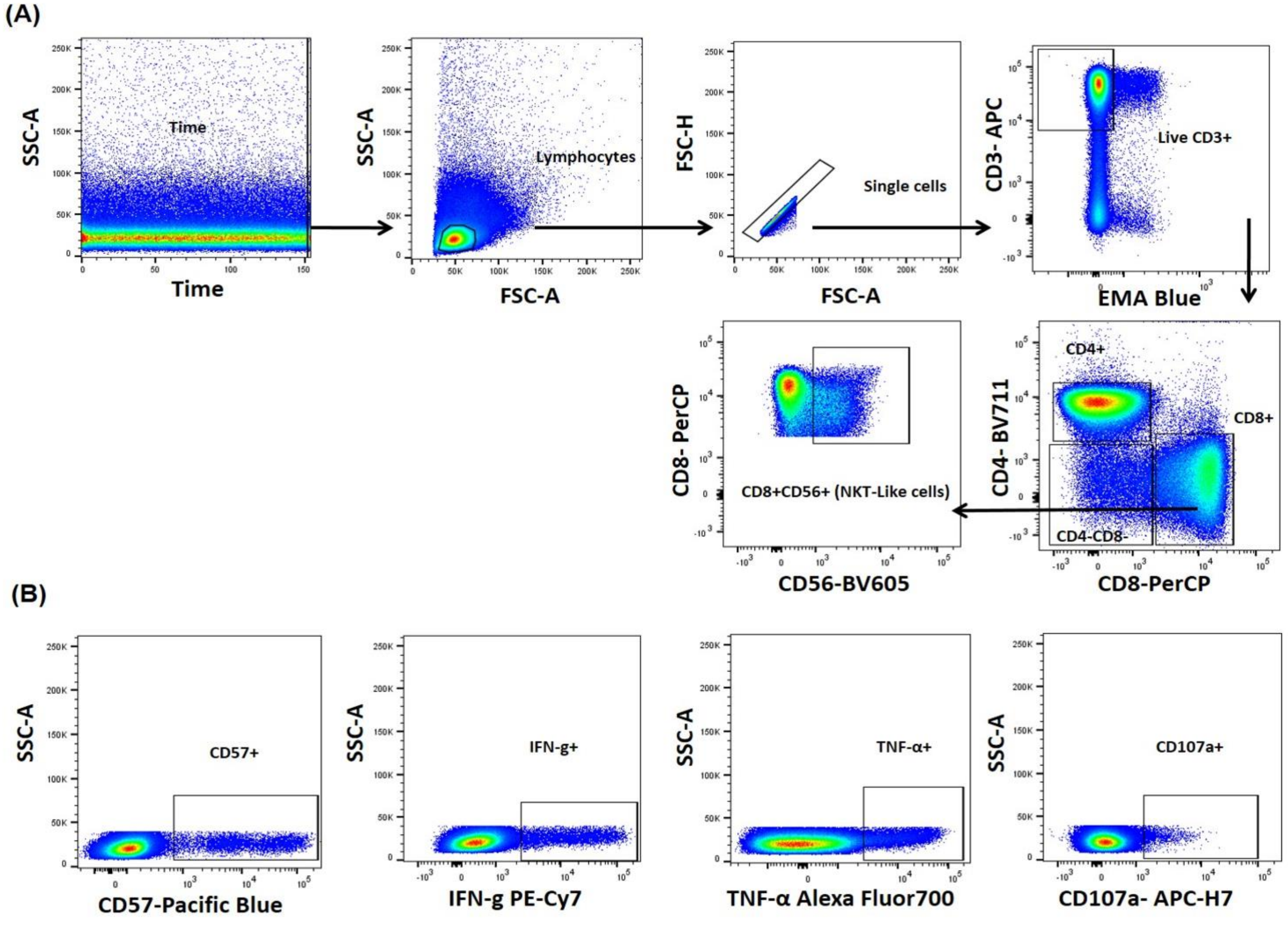

Figure S1. Flow cytometry gating strategy used in the analysis of T-cells. A) Time gate vs Side scatter dot blot (SSC-A) to detect differences in the flow, then peripheral blood lymphocytes (PBLs) were selected using forward (FSC) and side scatter (SSC) detectors. Live CD3+ T-cells were gated from PBLs after single cell gating, followed by identification of CD4+, CD8+, CD4-CD8- T-cells (DN: Double negative) and CD8+CD56+ T-cells (NKT-like cells). Arrows show the sequence of the gating used, starting from the Time gate. B) Single gates for CD107a, IFN-γ and TNF-α staining were determined within each T-cell subset on the basis of the unstimulated control. For CD57 expression, a Fluorescence minus one (FMO) control was used. Figure shows an example for stimulated CD8+ T-cells from a middle-aged CMV-seropositive individual.

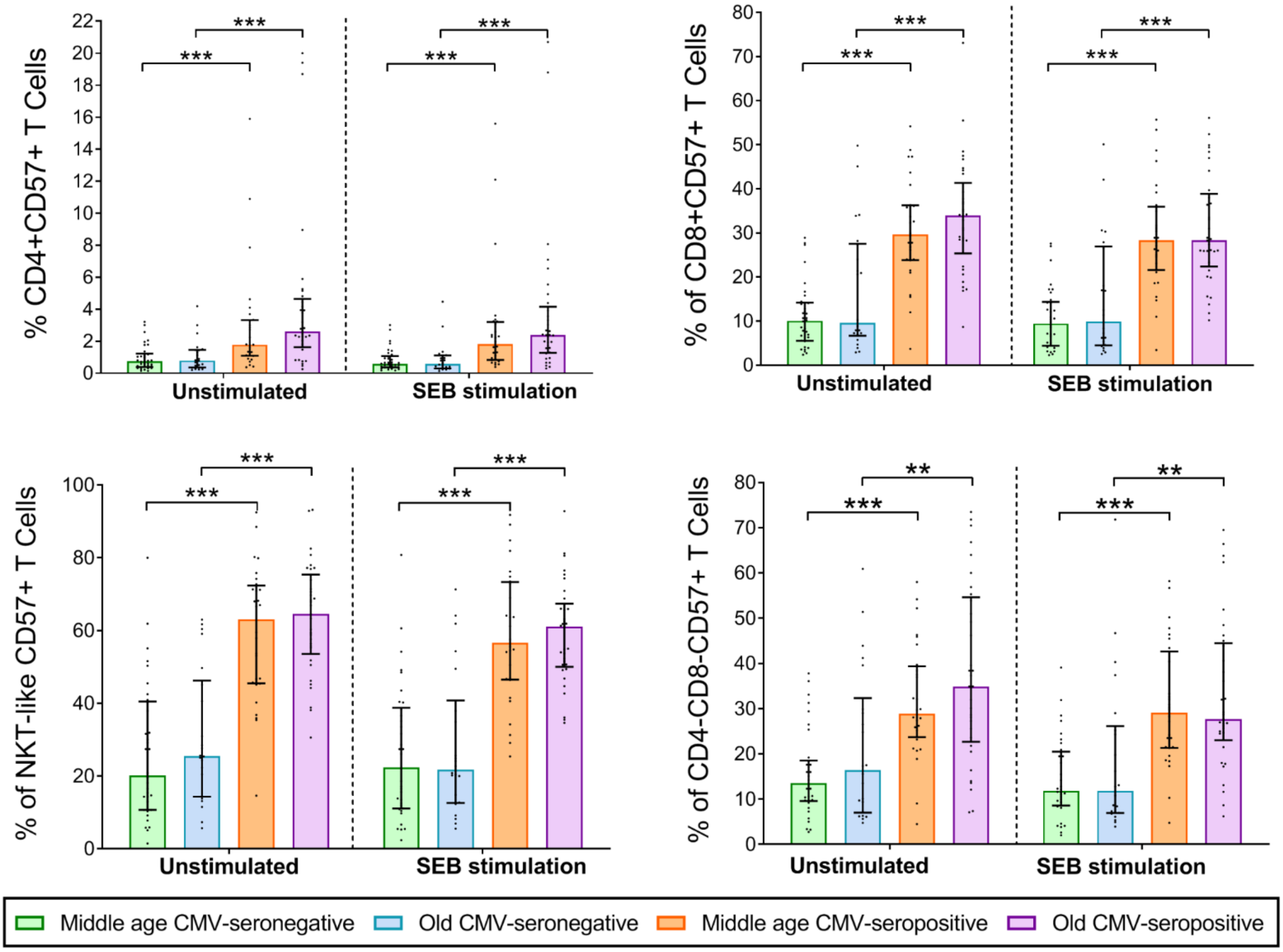

Figure S2. CD57 expression in T-cell subsets. Percentage of CD57+ T-cells within each subset studied among the four groups studied. Vertical black lines indicate interquartile ranges, ranging from the 25th to the 75th percentile. The median response for each category is indicated by the bars´ upper limit. Results were considered significant at $*p < 0.05$, $**p < 0.01$, and $***p < 0.001$.

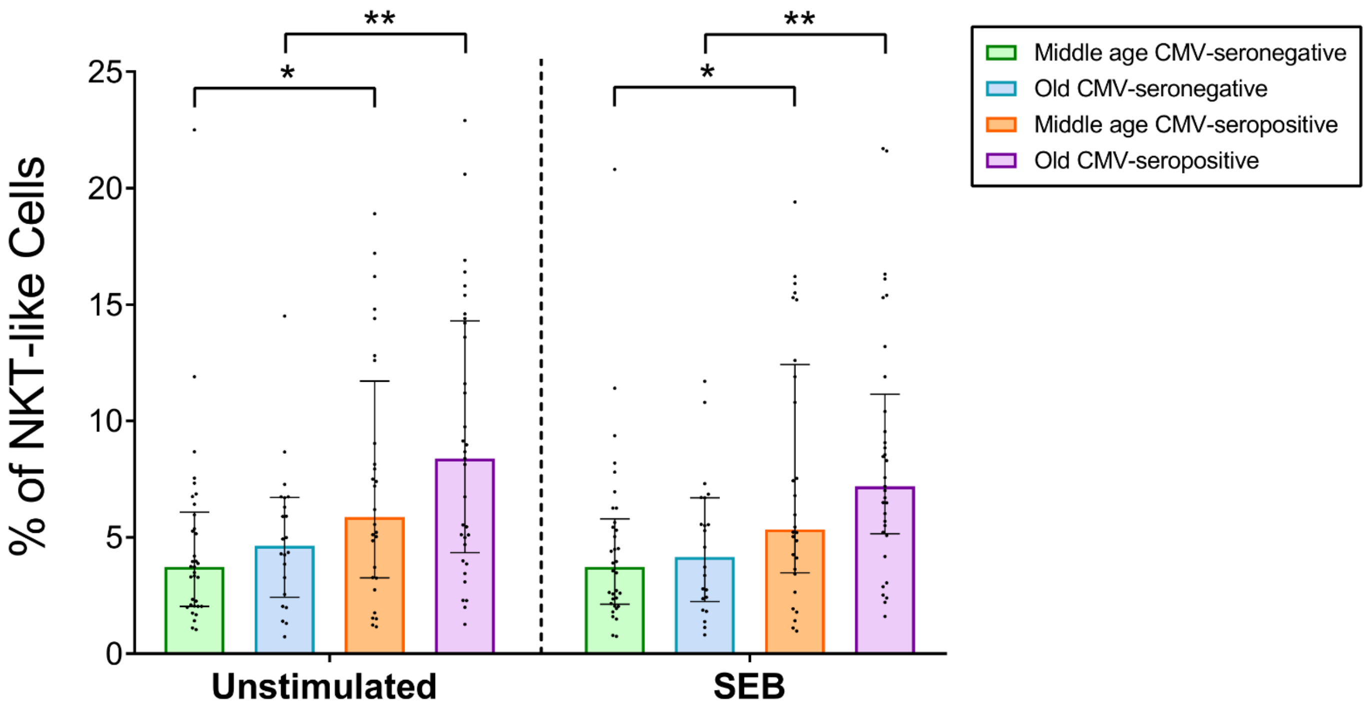

Figure S3. Percentage of NKT-like (CD8+ T-cells) in healthy individuals stratified by CMV serostatus and age. Percentage of NKT-like resting or SEB-stimulated T-cells among the four groups studied. Vertical black lines indicate interquartile ranges, ranging from the 25th to the 75th percentile. The median response for each category is indicated by the bars´ upper limit. Results were considered significant at **p* < 0.05, ***p* < 0.01, and ****p* < 0.001.

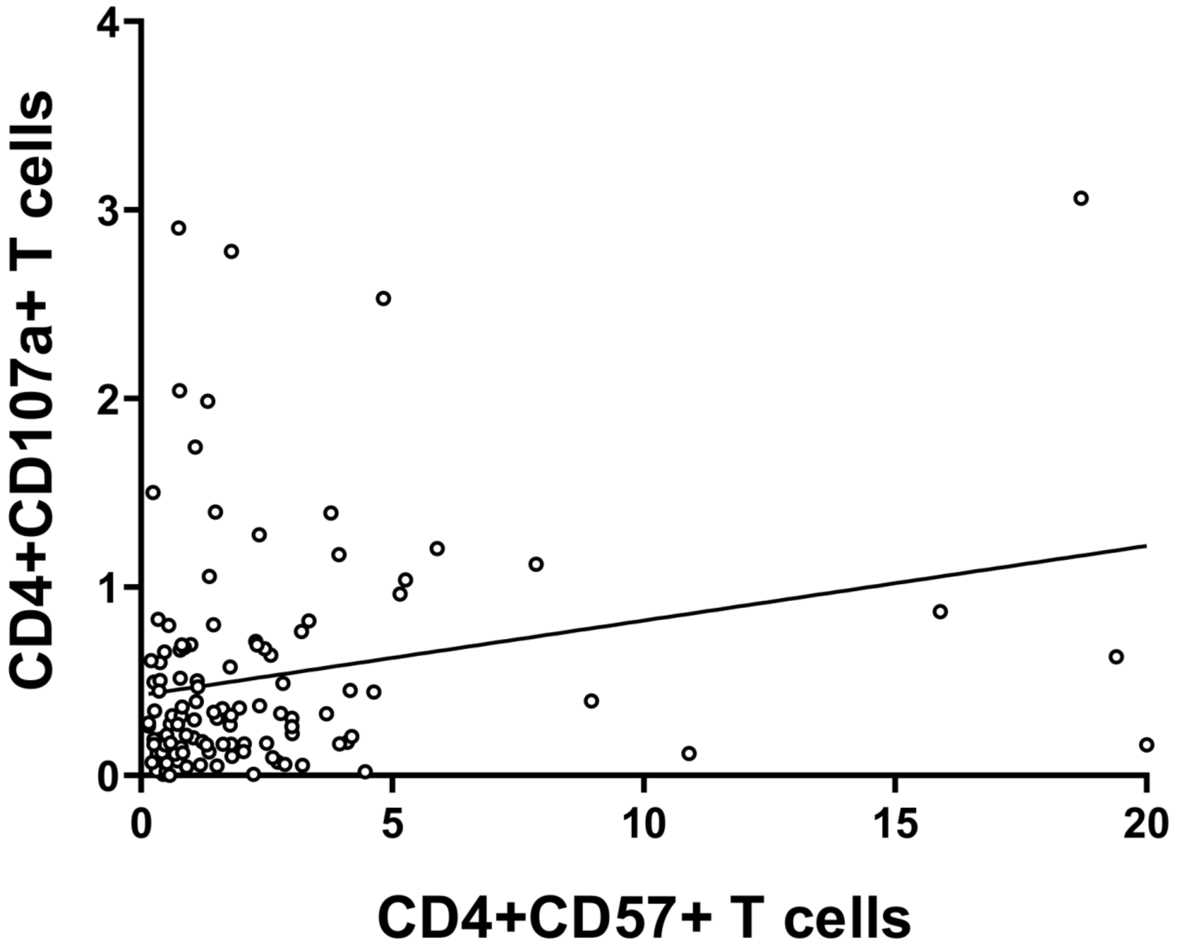

Figure S4. Spearman correlation between CD4+CD107+ and CD4+CD57+ T cells (p=0.03). Y axis represents the percentage of CD4+ T cells that express CD107a. X axis represents the percentage of CD4+CD57+ T cells.
